# Arthur Walbank

**Published:** 1937

**Authors:** 


### ARTHUR WALBANK, M.B., Ch.B. Leeds, F.R.C.S. Edin., D.O.M.S.*

MR. ARTHUR WALBANK, F.R.C.S. Edin., died at Clifton after a brief illness on January 28th, at the early age of thirty-eight. He was educated at Leeds, where he obtained the M.B. and Ch.B. degrees, and after holding resident posts there went to Bristol as House Surgeon to the Bristol Eye Hospital, which appointment he held from 1927 to 1930. After a further period of study he obtained the D.O.M.S. of the English Conjoint Board, and was admitted a Fellow of the Royal College of Surgeons of Edinburgh, and on returning to Bristol he was elected to the Honorary Staff of the Eye Hospital in 1931. During these few short years as Honorary Surgeon his whole-hearted co-operation in the work of the hospital had endeared him to his colleagues and patients alike. He was also Secretary to the South-Western Ophthalmological Society, and it was mainly due to his unflagging energy that these meetings were always valuable in discussion and cases. His keenness and efficiency in his work had already shown him to be an Ophthalmic Surgeon of great promise, and Bristol and the Bristol Eye Hospital are very much the poorer for the loss of Arthur Walbank at such an early stage in his career. He leaves a widow and one small, son, to whom our most sincere sympathy is extended.

* By courtesy of the Editor of the *British Medical Journal.*

